# Facile Preparation of CNT/Ag_2_S Nanocomposites with Improved Visible and NIR Light Photocatalytic Degradation Activity and Their Catalytic Mechanism

**DOI:** 10.3390/mi10080503

**Published:** 2019-07-30

**Authors:** Lijing Di, Tao Xian, Xiaofeng Sun, Hongqin Li, Yongjie Zhou, Jun Ma, Hua Yang

**Affiliations:** 1 College of Physics and Electronic Information Engineering, Qinghai Normal University, Xining 810008, China; 2 State Key Laboratory of Advanced Processing and Recycling of Non-ferrous Metals, Lanzhou University of Technology, Lanzhou 730050, China

**Keywords:** Ag_2_S nanoparticles, carbon nanotubes, CNT/Ag_2_S hybrid nanocomposites, photocatalysis

## Abstract

In this work, a series of carbon nanotubes (CNT)/Ag_2_S hybrid nanocomposites were successfully prepared by a facile precipitation method. Transmission electron microscope (TEM) observation indicates that Ag_2_S nanoparticles with an average particle size of ~25 nm are uniformly anchored on the surface of CNT. The photocatalytic activities of the CNT/Ag_2_S nanocomposites were investigated toward the degradation of rhodamine B (RhB) under visible and near-infrared (NIR) light irradiation. It is shown that the nanocomposites exhibit obviously enhanced visible and NIR light photocatalytic activities compared with bare Ag_2_S nanoparticles. Moreover, the recycling photocatalytic experiment demonstrates that the CNT/Ag_2_S nanocomposites possess excellent photocatalytic stability. The photoelectrochemical and photoluminescence measurements reveal the efficient separation of photogenerated charges in the CNT/Ag_2_S nanocomposites. This is the dominant reason behind the improvement of the photocatalytic activity. Based on active species trapping experiments, the possible photocatalytic mechanism of CNT/Ag_2_S nanocomposites for dye degradation under visible and NIR light irradiation was proposed.

## 1. Introduction

Recently, photocatalysis has come to be regarded as a promising technology to solve environmental pollution and energy problems [1,2,3]. As one of the typical photocatalysts, TiO_2_ can only respond to ultraviolet (UV) light (less than 5% solar energy) owing to its wide bandgap (~3.2 eV), which seriously limits its application in the photocatalytic field. It is well known that visible light and near-infrared light (NIR) accounts for about 48% and 46% of solar energy, respectively [4]. In view of the effective utilization of sunlight energy, much work has focused on the development of novel photocatalysts that can respond to visible light or NIR light [5,6,7,8].

Silver sulfide (Ag_2_S), as an important chalcogenide, has been widely studied due to its outstanding optical limiting properties, good chemical stability and potential applications in photoconductive cells, photovoltaic cells and superionic conductors [9,10]. More importantly, Ag_2_S exhibits a narrow bandgap (~1.0 eV) and a large absorption coefficient, which is very suitable for the effective absorption of visible light and NIR light. These prominent properties make it a promising candidate for the photodegradation of organic pollutants and water splitting under visible and NIR light irradiation [11,12,13,14,15]. On the other hand, because of its appropriate energy-band potentials, Ag_2_S has been widely employed as an ideal cocatalyst to combine with other photocatalysts, thus creating efficient composite photocatalysts, such as Ag_2_S/BiFeO_3_, Ag_2_S/BiVO_4_, Ag_2_S/ZnO, Ag_2_S/Bi_4_Ti_3_O_12_, Ag_2_S/SnS_2_, Ag_2_S/Co_3_O_4_, Ag_2_S/g-C_3_N_4_, Ag_2_S/ZnS, Ag_2_S/CQDs/CuBi_2_O_4_, Ag_2_S/Ag_3_PO_4_, Ag_2_S/Ag_2_CO_3_ and Ag_2_S/TiO_2_ [16,17,18,19,20,21,22,23,24,25,26,27]. Nevertheless, for the bare Ag_2_S, the high recombination rate of photogenerated electron-hole (e^−^-h^+^) pairs restricts its photocatalytic efficiency. As a result, many routes have been used to enhance the photocatalytic activity of Ag_2_S [28,29,30,31,32,33].

Carbon nanomaterials (e.g., carbon quantum dots (CQDs), carbon nanotubes (CNTs) and graphene) and noble metal nanoparticles (NPs) have a variety of intriguing physicochemical properties and offer a wide scope of technological applications in electronic devices, biomedicine, sensors, and wave absorption [34,35,36,37,38,39,40,41]. Moreover, due to their good carrier transport property, interesting photoluminescence (PL) up-conversion effect and localized surface plasmon resonance (LSPR) effect [42,43], these nanomaterials can also be used as excellent modifiers or co-catalysts to enhance the photocatalytic performances of semiconductor photocatalysts [31,32,33,44,45,46,47,48,49,50,51]. CNT can be assumed by folding single-layered graphene seamlessly into a 1D tubular structure. Owing to the unique properties of CNT, incorporation of CNT with photocatalysts is found to be an efficient route to enhance the photocatalytic activities of photocatalysts [44,45,46,47,48,49,50,51]. In CNT based composites, the photogenerated electrons of photocatalysts can readily migrate to the CNT, thus promoting the separation of photogenerated charges [44,45,46,47,48,49,50,51]. In previous work, Meng et al., had demonstrated the enhanced visible-light-driven photocatalytic activity of CNT/Ag_2_S towards the degradation of Texbrite BA-L (TAB) [33]. However, the photocatalytic activity of CNT/Ag_2_S for the degradation of dyes under NIR light irradiation and the corresponding photocatalytic mechanism were rarely investigated. To gain insight into the photocatalytic application of CNT/Ag_2_S hybrid photocatalyst, further investigation of its NIR photocatalytic performance and mechanism is still necessary.

In this work, we prepared CNT/Ag_2_S nanocomposites through a facile precipitation method. Compared with the sintering process and hydrothermal route, this method does not need complex technological processes, such as heat treatment and hydrothermal conditioning [22,27]. The photocatalytic activities of the photocatalyst toward the degradation of rhodamine B (RhB) under visible and NIR light irradiation were investigated in detail, and the photocatalytic mechanism was proposed. We propose that this work will offer an efficient modification method for the improvement of visible and NIR light photocatalytic activity of Ag_2_S nanoparticles.

## 2. Materials and Methods

### 2.1. Fabrication of Ag_2_S Nanoparticles

The Ag_2_S nanoparticles were prepared by a precipitation method. AgNO_3_ (2 mmol) was added into distilled water (30 mL) under magnetic stirring (pH ~6.1). Na_2_S (1 mmol) was introduced into distilled water (20 mL) to form a homogeneous solution (pH ~11.7). After that, Na_2_S solution was added drop by drop into AgNO_3_ solution under vigorous magnetic stirring for 5 h, during which a black suspension was obtained (pH ~5.8). During the preparation process, the pH value of the solution was maintained at a natural condition. The obtained black product was separated by centrifugation, washed with distilled water several times, and then dried in an oven at 60 °C for 6 h.

### 2.2. Fabrication of Carbon Nanotubes (CNT)/Ag_2_S Nanocomposites

**Scheme 1:**
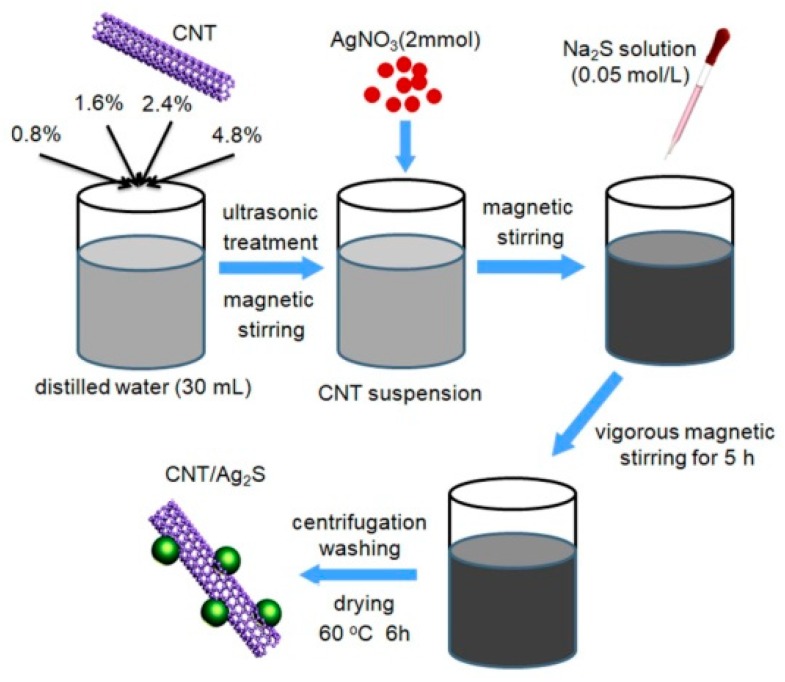
The schematic illustration of preparation process for carbon nanotubes (CNT)/Ag_2_S nanocomposite.

For the preparation of CNT/Ag_2_S nanocomposites (Scheme 1), a certain amount of multi-walled CNT, purchased from Nanjing XFNano Materials Tech Co. Ltd. (Nanjing, China), was introduced into distilled water (30 mL) with ultrasonic treatment and magnetic stirring to obtain a homogeneous suspension. Subsequently, AgNO_3_ (2 mmol) was dissolved into the CNT suspension under magnetic stirring (pH ~4.1). On the other hand, Na_2_S (1 mmol) was added into distilled water (20 mL) to form a uniform solution (pH ~11.8). During the above process, the pH value of the solution was maintained at a natural condition. The following precipitate process and washing/drying procedure was similar to that for Ag_2_S preparation. To investigate the effect of CNT content on the photocatalytic activity of CNT/Ag_2_S nanocomposites, a series of CNT/Ag_2_S nanocomposites with different mass contents of CNT (0.8%, 1.6%, 2.4% and 4.8%) were prepared. These composite samples were correspondingly termed as 0.8% CNT/Ag_2_S, 1.6% CNT/Ag_2_S, 2.4% CNT/Ag_2_S and 4.8% CNT/Ag_2_S.

### 2.3. Photocatalytic Activity Test

The photocatalytic activities of the photocatalyst were investigated toward the degradation of RhB separately under illumination of visible light (300 W xenon lamp with a 420 nm cut-off filter) and NIR light (300 W xenon lamp with an 800 nm cut-off filter). Typically, 0.1 g sample was added into 200 mL RhB solution (concentration: 5 mg L^−1^). Before photocatalytic reaction, the above mixture was magnetically stirred in the dark for 0.5 h to achieve adsorption-desorption equilibrium between the photocatalyst and RhB molecules. During the photocatalytic process, a small volume of the reaction solution was sampled at a given time interval and centrifuged to separate the photocatalyst. The concentration of RhB solution was measured by detecting the absorbance of the obtained supernate at a wavelength of ~554 nm using an ultraviolet-visible (UV-vis) spectrophotometer. The photocatalytic stability of the photocatalyst was evaluated by recycling degradation experiment. After each photocatalytic reaction, the photocatalyst was collected and recovered by washing and drying. The recovered photocatalyst was introduced into the new RhB solution for the next photocatalytic experiment under the same condition. To detect the active species responsible for dye degradation, the active species trapping experiment was carried out. Ethanol (10% by volume) and ethylene diamine tetraacetic acid (EDTA, 2 mM) were employed as the scavengers of hydroxyl (•OH) and photogenerated holes (h^+^), respectively. The trapping experimental process was performed under the same conditions as described for the above photocatalytic procedure. To examine the role of superoxide (•O_2_^−^) and hydrogen peroxide (H_2_O_2_), N_2_ gas was bubbled into the reaction solution to expel the dissolved O_2_ and thus prevent the generation of •O_2_^−^ and H_2_O_2_.

### 2.4. Characterization

The phase purity of the photocatalyst was detected by X-ray diffractometer (XRD) (Bruker AXS, Karlsruhe, Germany). The morphology observation of the photocatalyst was performed by a field-emission scanning electron microscope (SEM) (JEOL Ltd., Tokyo, Japan) and field emission transmission electron microscope (TEM) (JEOL Ltd., Tokyo, Japan). The composition and surface chemical states of the photocatalyst were investigated though X-ray photoelectron spectroscopy (XPS) (Physical Electronics, Chanhassen, MN, USA). The UV-vis diffuse reflectance spectra of the photocatalyst were measured using a UV-vis spectrophotometer with BaSO_4_ as a reference (Beijing Purkinje General Instrument Co. Ltd., Beijing, China). The photoluminescence (PL) spectra of the photocatalyst were obtained on a fluorescence spectrophotometer with the excitation wavelength of ~265 nm (Shimadzu RF-6000, Kyoto, Japan). Photoelectrochemical measurements were performed on a CHI 660C electrochemical workstation (Shanghai Chenhua Instrument Co. Ltd, Shanghai, China) equipped with a three-electrode cell configuration. The working electrode preparation and measurement procedures were the same as those reported in the literature [52]. The used electrolyte was 1 mol L^−1^ Na_2_SO_4_ aqueous solution, and the light source was a 300 W xenon lamp with a 420 nm cut-off filter.

## 3. Results and Discussion

### 3.1. X-Ray Diffractometer (XRD) Analysis

**Figure 1:**
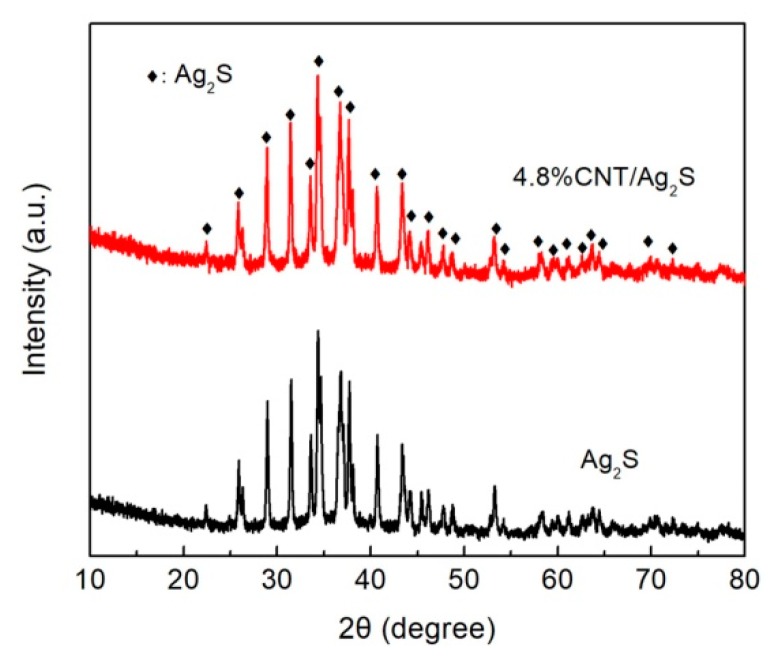
X-ray diffractometer (XRD) patterns of Ag_2_S nanoparticles and the 4.8% CNT/Ag_2_S composite.

The XRD patterns of bare Ag_2_S nanoparticles and the 4.8% CNT/Ag_2_S nanocomposite are shown in Figure 1. For the Ag_2_S nanoparticles, all of the diffraction peaks can be indexed to the monoclinic structure of Ag_2_S (JCPDS Card No. 14-0072). Notably, the XRD pattern of 4.8% CNT/Ag_2_S is very similar to that of the Ag_2_S nanoparticles and no trace of other impurities is detected, suggesting that the phase structure of Ag_2_S does not undergo obvious change when coupled with CNT. In addition, no characteristic diffraction peaks of CNT are observed in the XRD pattern of the nanocomposite, which is mainly due to its low content and weak diffraction intensity [48].

### 3.2. Optical Absorption Properties

**Figure 2:**
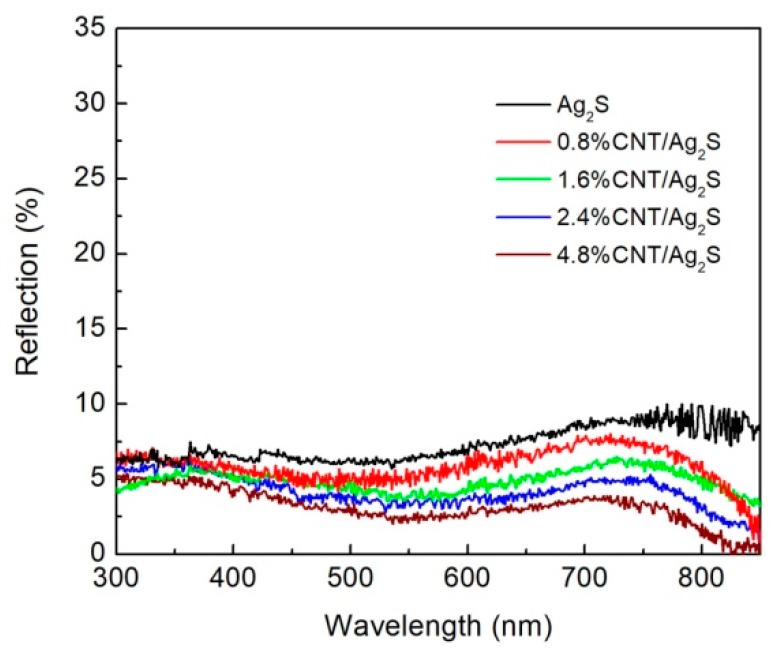
Ultraviolet-visible (UV-visible) diffuse reflectance spectra of Ag_2_S nanoparticles and the CNT/Ag_2_S nanocomposites.

It is noted that the optical absorption properties of semiconductors have an important effect on their performances, which can be determined by UV-vis diffuse reflectance spectra (DRS) measurements [53,54]. The DRS spectra of Ag_2_S nanoparticles and CNT/Ag_2_S nanocomposites with different CNT contents are displayed in Figure 2. It can be seen that bare Ag_2_S nanoparticles exhibit strong light absorption in the entire range of the UV-vis light region. The combination of CNT with Ag_2_S nanoparticles leads to an enhanced light absorption in the whole wavelength range. Moreover, the absorption intensity of the CNT/Ag_2_S photocatalyst increases with increasing CNT content.

### 3.3. Morphology Observations

**Figure 3:**
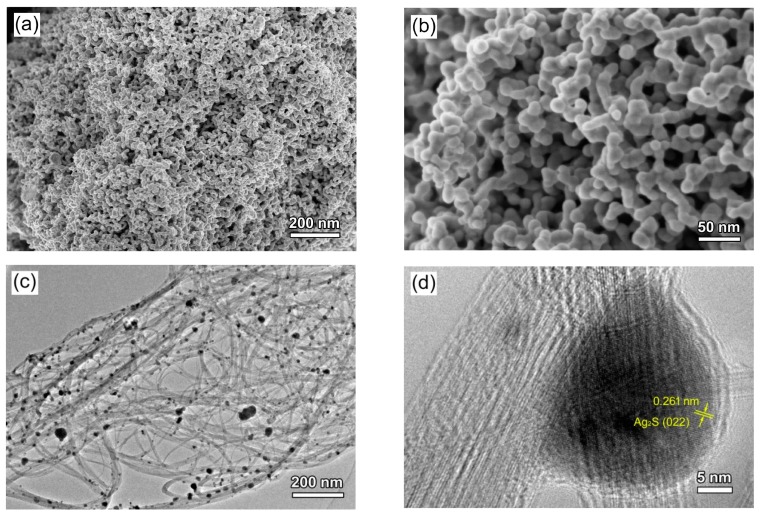
(**a**,**b**) scanning electron microscope (SEM) image of Ag_2_S nanoparticles; (**c**) transmission electron microscope (TEM) and (**d**) high resolution TEM (HRTEM) image of 4.8% CNT/Ag_2_S.

Figure 3a,b present the SEM images of Ag_2_S nanoparticles, indicating that Ag_2_S is crystallized into sphere-like particles with an average diameter of ~25 nm. Figure 3c,d show the TEM image and high-resolution TEM (HRTEM) image of the 4.8% CNT/Ag_2_S photocatalyst, revealing that Ag_2_S nanoparticles are uniformly anchored with CNT. The HRTEM image clearly depicts the multi-walled characteristic of CNT and obvious lattice fringes of Ag_2_S nanoparticles. The interplanar spacing of ~0.261 nm can be assigned to the (022) planes of Ag_2_S. The good coupling of Ag_2_S nanoparticles with CNT is beneficial for the migration of photogenerated electrons from Ag_2_S nanoparticles to CNT.

**Figure 4:**
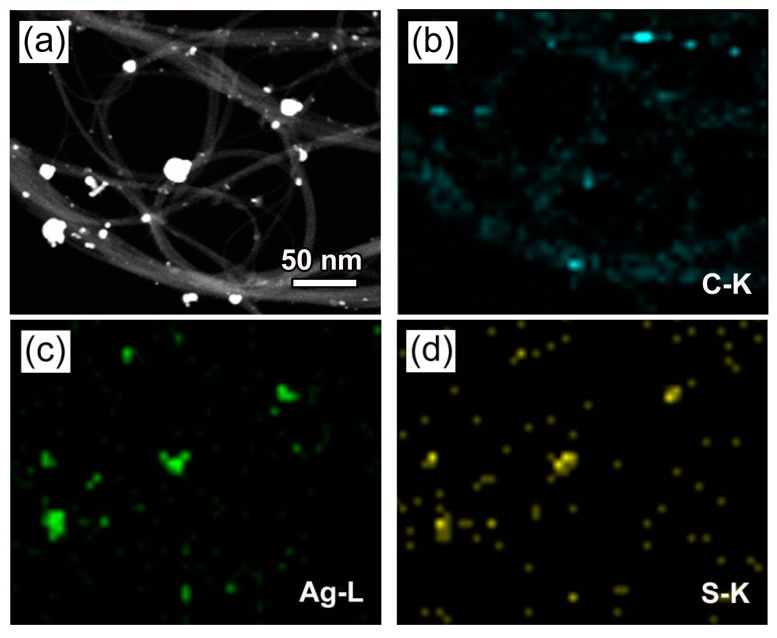
(**a**) Dark field scanning TEM (DF-STEM) image of 4.8% CNT/Ag_2_S. (**b**–**d**) The corresponding energy dispersive X-ray elemental mapping images in the region of (**a**).

Elemental mapping is an important method to observe the elemental distribution and microstructure of materials [55]. Figure 4a presents the dark field scanning TEM (DF-STEM) image of 4.8% CNT/Ag_2_S, and the corresponding elemental maps are shown in Figure 4b–d. It is seen that the CNT presents the elemental distribution of C, and the anchored nanoparticles consist of Ag and S elements. This indicates the formation of a CNT/Ag_2_S hybrid structure.

### 3.4. X-ray Photoelectron Spectroscopy (XPS) Analysis

**Figure 5:**
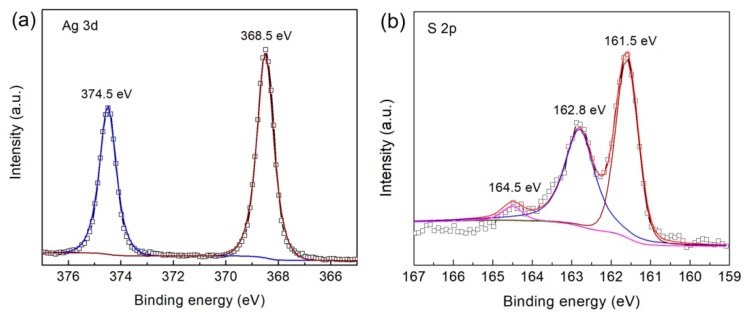

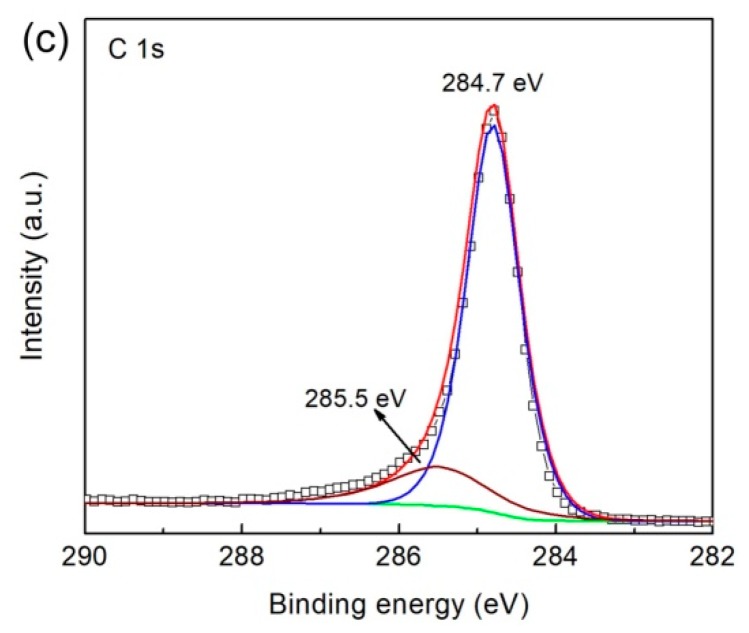
High-resolution X-ray photoelectron spectroscopy (XPS) spectra of 4.8% CNT/Ag_2_S: (**a**) Ag 3d, (**b**) S 2p and (**c**) C 1s.

The surface chemical states and elemental composition of 4.8% CNT/Ag_2_S were detected by XPS, as depicted in Figure 5. In the spectrum of Ag 3d (Figure 5a), the core-electron binding energies of Ag 3d_3/2_ and Ag 3d_5/2_ are found at 374.5 and 368.5 eV, respectively, which correspond to the characteristic peaks of Ag^+^ [56]. The S 2p spectrum (Figure 5b) displays two obvious fitted peaks at 162.8 and 161.5 eV, which are attributed to S 2p_1/2_ and S 2p_3/2_ of S^2−^, respectively. In addition, the weak peak located at ~164.5 eV belongs to the satellite peak of S 2p_1/2_, which is consistent with previous report [57]. The deconvoluted spectrum of C 1s is presented in Figure 5c, where the peak at 284.7 eV is assigned to the sp^2^ hybridized carbon (C-C) and the peak at 285.5 eV is ascribed to the defect of hybridized carbon [58].

### 3.5. Photocatalytic Activities

**Figure 6:**
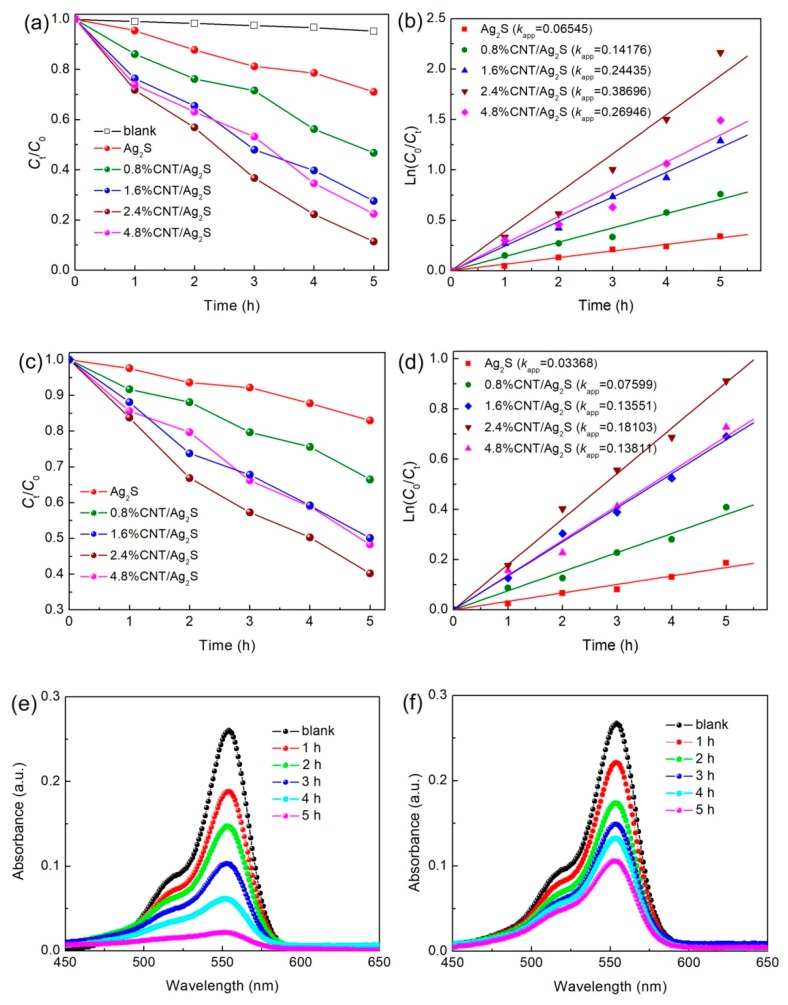
(**a**) Photocatalytic activities and (**b**) kinetic fit plots of bare Ag_2_S and CNT/Ag_2_S namocomposites for the degradation of rhodamine B (RhB) under visible light irradiation; (**c**) Photocatalytic activities and (**d**) kinetic fit plots of the samples under near-infrared (NIR) light irradiation; (**e**,**f**) The absorption spectra of RhB solution over 2.4% CNT/Ag_2_S under visible and NIR light irradiation, respectively.

The visible and NIR light photocatalytic activities of the CNT/Ag_2_S photocatalyst were assessed by the degradation of RhB. Figure 6a presents the concentration change of RhB photocatalyzed by the samples with visible light illumination. For comparison, the blank experiment was carried out without the addition of photocatalysts. Only a slight decrease in RhB concentration is observed after 5 h illumination, suggesting that the self-degradation of RhB under visible light irradiation can be neglected. When Ag_2_S nanoparticles are used as the photocatalyst, about 28.9% of RhB is degraded after visible light irradiation for 5 h, indicating that Ag_2_S nanoparticles exhibit weak photocatalytic activity. This phenomenon is probably attributed to the high recombination rate of photogenerated charges in pure Ag_2_S [11,12,13]. The combination of CNT with Ag_2_S nanoparticles leads to an obvious enhancement in the photocatalytic activity of Ag_2_S nanoparticles. After 5 h exposure, the degradation percentage of the dye over 0.8% CNT/Ag_2_S, 1.6% CNT/Ag_2_S, 2.4% CNT/Ag_2_S and 4.8% CNT/Ag_2_S is observed as ~53.2%, ~72.4%, ~88.5% and ~77.5%, respectively. It is found that when the content of CNT is increased from 0.8% to 2.4%, the photodegradation percentage of the dye is gradually raised. The 2.4% CNT/Ag_2_S composite exhibits the optimal degradation percentage. However, further increasing the content of CNT leads to the decreased photoactivity of the resultant composites. This could be attributed to the fact that the excessive CNT may shield the light, thus decreasing the photon absorption of the photocatalysts [48]. The photocatalytic reaction process can be fitted by the first-order kinetic equation Ln (*C*_0_/*C*_t_) = *k*_app_*t* [59], as shown in Figure 6b. It is found that the 2.4% CNT/Ag_2_S composite exhibits the highest apparent first-order reaction rate constant, *k*_app_, which is about 5.9 times higher than that for bare Ag_2_S nanoparticles. Compared with graphene-modified Ag_2_S, the visible-light-driven photocatalytic activity Ag_2_S can be much improved by the decoration of CNT [32].

Figure 6c displays the variation of RhB concentration as a function of NIR light irradiation over the samples. It is seen that the samples also manifest an important NIR photodegradation activity. Photocatalyzed by the optimal 2.4% CNT/Ag_2_S composite with 5 h of NIR light exposure, ~59.8% of RhB is observed to be degraded. Photodegradation kinetics analysis, as shown in Figure 6d, implies that the 2.4% CNT/Ag_2_S composite has a NIR light photocatalytic activity about 5.4 times higher than that of bare Ag_2_S nanoparticles. Figure 6e,f illustrate the time-dependent absorption spectra of the RhB solution photocatalyzed by 2.4% CNT/Ag_2_S with irradiation of visible light and NIR light, respectively. The decreased absorption intensity of the RhB solution with irradiation time further confirms the photodegradation of RhB under both visible and NIR light irradiation.

**Figure 7:**
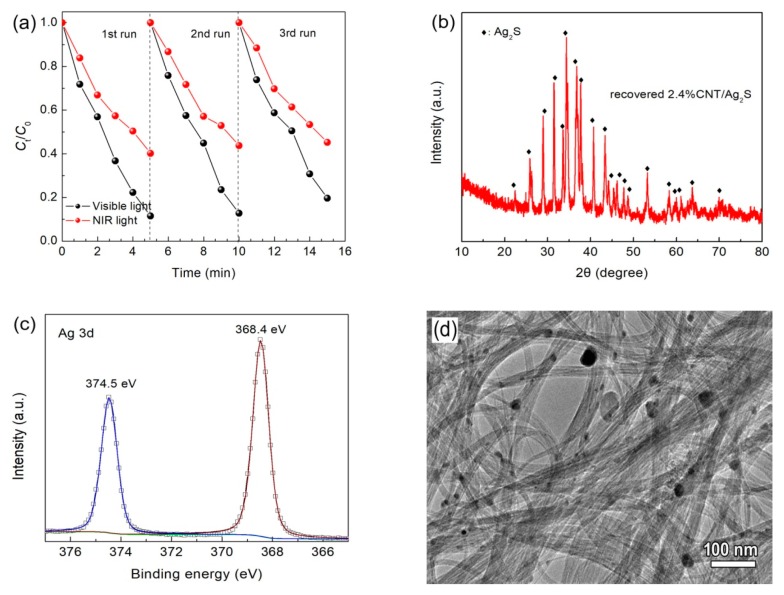
(**a**) Photocatalytic degradation of RhB over 2.4% CNT/Ag_2_S during three cycles under visible and NIR light irradiation; (**b**) XRD pattern, (**c**) Ag 3d XPS spectrum and (**d**) TEM image of photocatalytically used 2.4% CNT/Ag_2_S.

In addition to the photocatalytic activity, recyclability of photocatalysts is considered to be another important criterion for their practical application. The reusability of 2.4% CNT/Ag_2_S was examined by the recycling photocatalytic degradation of RhB separately under visible and NIR light irradiation. As shown in Figure 7a, after three consecutive cycles of photocatalytic reaction, no obvious decrease of the RhB degradation percentage is detected. To further evaluate the structural and morphological stability of 2.4% CNT/Ag_2_S after recycling photocatalytic reaction, the TEM observation, XPS detection and XRD characterization were carried out. The XRD pattern in Figure 7b demonstrates that Ag_2_S undergoes no detectable structural change and remains the monoclinic structure. The XPS spectrum of Ag 3d for the recovered 2.4% CNT/Ag_2_S photocatalyst (Figure 7c) suggests that the Ag_2_S in the composite is stable without being reduced after consecutive photocatalytic reaction, and the similar report can be found in other literature [60]. The TEM image (Figure 7d) shows that Ag_2_S nanoparticles are still well anchored onto CNT, and no obvious exfoliate phenomenon is observed. The above results reveal that the CNT/Ag_2_S photocatalyst exhibits good photocatalytic and structural stability.

### 3.6. Photogenerated Charge Behavior

**Figure 8:**
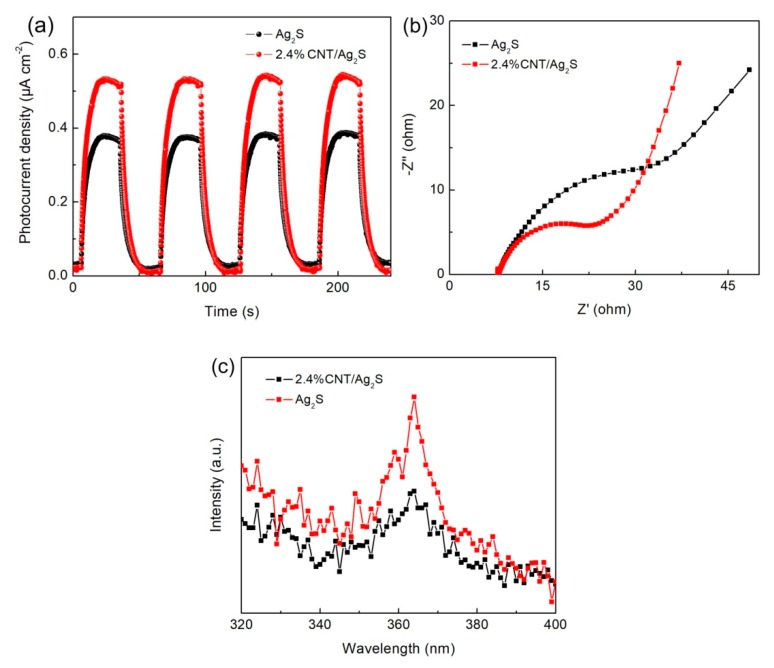
(**a**) Photocurrent response plots, (**b**) electrochemical impedance spectroscopy (EIS) spectra and (**c**) photoluminescence (PL) spectra of Ag_2_S and 2.4% CNT/Ag_2_S.

It is well known that the photocatalytic activity of photocatalysts is highly related to their photogenerated charges behavior. Transient photocurrent response, electrochemical impedance spectroscopy (EIS) and PL spectroscopy are useful methods to investigate the separation behavior of photoinduced charges [61,62]. Figure 8a presents the photocurrent response plots of Ag_2_S and 2.4% CNT/Ag_2_S under intermittent visible light irradiation. It can be seen that both the samples exhibit fast photocurrent responses with on-off cycles. The photocurrent value of 2.4% CNT/Ag_2_S is much higher than that of bare Ag_2_S, indicating that the introduction of CNT promotes the separation of photogenerated electrons and holes in Ag_2_S. Figure 8b shows the EIS spectra of the samples, indicating that 2.4% CNT/Ag_2_S exhibits a smaller impedance arc radius than that of bare Ag_2_S. This reveals a higher efficiency of charge transfer in the CNT/Ag_2_S photocatalyst. The PL spectra of Ag_2_S and 2.4% CNT/Ag_2_S are shown in Figure 8c. An obvious emission peak is observed at ~363 nm for both the samples, which is probably attributed to the recombination of photogenerated charges. The 2.4% CNT/Ag_2_S photocatalyst exhibits a relatively weak PL emission peak compared with bare Ag_2_S, which further confirms the efficient separation of photogenerated charges in the composite.

### 3.7. Photocatalytic Mechanisms

**Figure 9:**
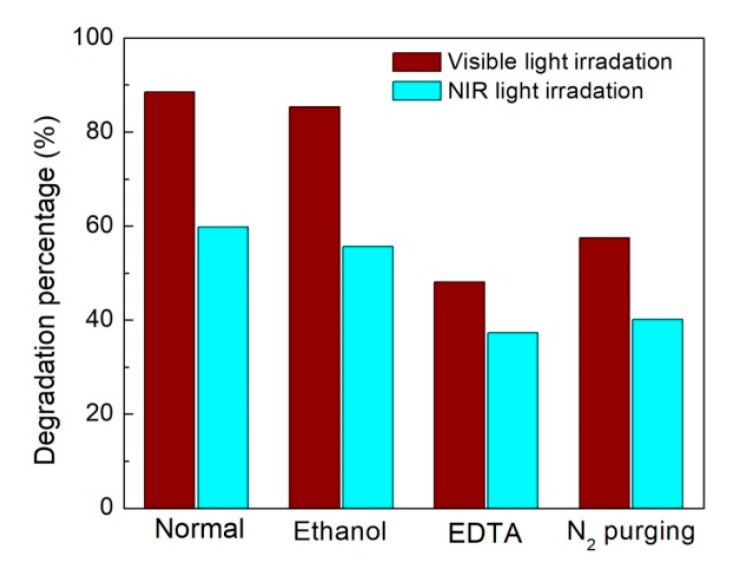
Effects of ethylene diamine tetraacetic acid (EDTA), ethanol and N_2_ purging on the photocatalytic degradation of rhodamine B (RhB) over 2.4% CNT/Ag_2_S under both visible and NIR light irradiation.

To clarify the photocatalytic mechanism of the CNT/Ag_2_S photocatalyst, active species trapping experiments were performed to determine the main active species involved in the photocatalytic reaction, as shown in Figure 9. Under visible light irradiation, the introduction of ethanol results in a slight decrease of degradation percentage, suggesting that •OH plays a minor role in the photocatalytic degradation of RhB. In contrast, the photocatalytic degradation of RhB obviously decreases with the addition of EDTA, which indicates that h^+^ is the major active species responsible for the degradation of the dye. •O_2_^−^ and H_2_O_2_, generally generated from the reaction between photogenerated electrons and O_2_, could also be the active species in the photocatalytic reaction. The N_2_ purging can expel O_2_ dissolved in the solution and inhibit the generation of •O_2_^−^ or H_2_O_2_. It is found that the degradation of RhB is remarkably suppressed after N_2_ purging, revealing that •O_2_^−^ and/or H_2_O_2_ play an important role in the photocatalytic reaction. Under NIR light irradiation, a similar active species trapping behavior is observed.

**Figure 10:**
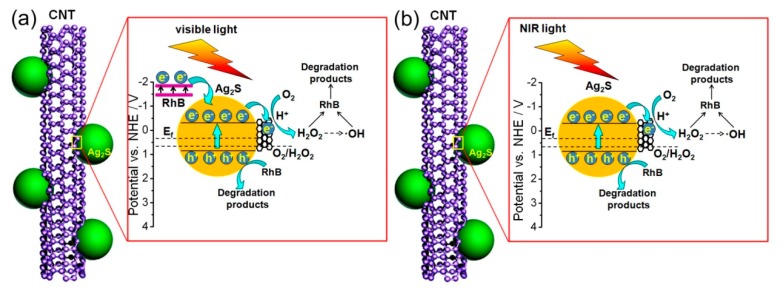
The proposed photocatalytic mechanism of the CNT/Ag_2_S photocatalyst towards the degradation of the dye under (**a**) visible and (**b**) NIR light irradiation.

The possible photocatalytic mechanism of the CNT/Ag_2_S photocatalyst is presented in Figure 10. When the reaction system is irradiated by visible light (Figure 10a), the Ag_2_S nanoparticles are excited to generate electrons and holes. It is noted that the RhB molecule can also absorb visible light in the wavelength range from 500 to 600 nm (Figure 6e,f), and therefore its photosensitization effect during the visible-light photocatalytic process should be considered. In this photosensitization process, the photogenerated electrons from excited RhB molecule will transfer to the conduction band (CB) of Ag_2_S because the redox potential of RhB (−1.42 V vs. normal hydrogen electrode (NHE)) is negative to the CB potential of Ag_2_S (−0.3 V vs. NHE) [63]. On the other hand, Under NIR light irradiation (Figure 10b), only Ag_2_S nanoparticles can be excited, leading to the generation of electrons and holes. Whether irradiated by visible or NIR light, the recombination rate of photogenerated charges in Ag_2_S is high, and thus only a small fraction of them participate in the photocatalytic reaction. It is demonstrated that CNT can act as an excellent electron acceptor due to its efficient electron conductivity [37]. Therefore, after the formation of hybrid structures between Ag_2_S nanoparticles and CNT, the photogenerated electrons in Ag_2_S can easily migrate to CNT, which inhibits the recombination of photogenerated charges and leads to the enhancement of photocatalytic activity. This charge migration process is feasible from a thermodynamic point of view because the Fermi level of CNT (+0.44 V vs. NHE) is positive to the CB potential of Ag_2_S (−0.3 V vs. NHE) [64,65]. The photogenerated electrons in the Fermi level of CNT cannot reduce O_2_ to •O_2_^−^ (*E*^0^(O_2_/•O_2_^−^) = −0.13 V vs. NHE) [66], but they can reduce O_2_ to produce H_2_O_2_ (*E*^0^(O_2_/H_2_O_2_) = +0.695 vs. NHE) [67]. This confirms that H_2_O_2_ is one of the active species responsible for the degradation of the dye, which explains the inhibition phenomenon of the dye degradation after N_2_ purging. Furthermore, a portion of H_2_O_2_ could transform into •OH through a series of reactions [65], which is considered to be the major route for the generation of •OH in this reaction. On the other hand, compared with the redox potential of OH^−^/•OH (+1.89 V vs. NHE), the photogenerated holes in the VB of Ag_2_S (+0.7 V vs. NHE) is not positive enough to oxidize OH^−^ to •OH [65,68]. However, the photogenerated holes in Ag_2_S can directly oxide the dye, as confirmed by the active species trapping experiment.

## 4. Conclusions

A series of CNT/Ag_2_S photocatalyst with different contents of CNT have been successfully synthesized through a simple precipitation method. It is found that the CNT/Ag_2_S photocatalyst exhibit obviously enhanced visible and NIR light photocatalytic activity for the degradation of RhB when compared with bare Ag_2_S nanoparticles. Moreover, the CNT/Ag_2_S photocatalysts are demonstrated to be stable visible and NIR light photocatalysts. The enhanced photocatalytic activity of the CNT/Ag_2_S photocatalyst is mainly attributed to the excellent electron-accepting ability of CNT, which can serve as an electron trap to promote the separation of photogenerated charges in Ag_2_S nanoparticles. The results of this study provide an efficient modification route for the enhancement of visible and NIR light photocatalytic activity of Ag_2_S nanoparticles, which is beneficial for their practical applications in the photocatalytic field.

## References

[1] Fox M.A., Dulay M. (1993). Heterogeneous Photocatalysis. Chem. Rev..

[2] Kudo A., Miseki Y. (2009). Heterogeneous photocatalyst materials for water splitting. Chem. Soc. Rev..

[3] Yan Y.X., Yang H., Zhao X.X., Zhang H.M., Jiang J.L. (2018). A hydrothermal route to the synthesis of CaTiO_3_ nanocuboids using P25 as the titanium source. J. Electron. Mater..

[4] Tian J., Leng Y.H., Zhao Z.H., Xia Y., Sang Y.H., Hao P., Zhan J., Li M.C., Liu H. (2015). Carbon quantum dots/hydrogenated TiO_2_ nanobelt heterostructures and their broad spectrum photocatalytic properties under UV, visible, and near-infrared irradiation. Nano Energy.

[5] Di L.J., Yang H., Xian T., Chen X.J. (2018). Facile synthesis and enhanced visible-light photocatalytic activity of novel p-Ag_3_PO_4_/n-BiFeO_3_ heterojunction composites for dye degradation. Nanoscale Res. Lett..

[6] Ye Y.C., Yang H., Zhang H.M., Jiang J.L. (2018). A promising Ag_2_CrO_4_/LaFeO_3_ heterojunction photocatalyst applied to photo-Fenton degradation of RhB. Environ. Technol..

[7] Jiang W., Wang X.Y., Wu Z.M., Yue X.N., Yuan S.J., Lu H.F., Liang B. (2015). Silver Oxide as Superb and Stable Photocatalyst under Visible and Near-Infrared Light Irradiation and Its Photocatalytic Mechanism. Ind. Eng. Chem. Res..

[8] Wei N., Cui H.Z., Song Q., Zhang L.Q., Song X.J., Wang K., Zhang Y.F., Li J., Wen J., Tian J. (2016). Ag_2_O nanoparticle/TiO_2_ nanobelt heterostructures with remarkable photo-response and photocatalytic properties under UV, visible and near-infrared irradiation. Appl. Catal. B Environ..

[9] Shen H.P., Jiao X.J., Oron D., Li J.B., Lin H. (2013). Efficient electron injection in non-toxic silver sulfide (Ag_2_S) sensitized solar cells. J. Power Sources.

[10] Wu J.J., Chang R.C., Chen D.W., Wu C.T. (2012). Visible to near-infrared light harvesting in Ag_2_S nanoparticles/ZnO nanowire array photoanodes. Nanoscale.

[11] Cao Q., Che R.C., Chen N. (2014). Facile and rapid growth of Ag_2_S microrod arrays as efficient substrates for both SERS detection and photocatalytic degradation of organic dyes. Chem. Commun..

[12] Huo P.W., Liu C.Y., Wu D.Y., Guan J.R., Li J.Z., Wang H.Q., Qi T., Li X.Y., Yan Y.S., Yuan S.Q. (2018). Fabricated Ag/Ag_2_S/reduced grapheme oxide composite photocatalysts for enhancing visible light photocatalytic and antibacterial activity. J. Ind. Eng. Chem..

[13] Pourahmad A. (2012). Ag_2_S nanoparticle encapsulated in mesoporous material nanoparticles and its application for photocatalytic degradation of dye in aqueous solution. Superlattices Microstruct..

[14] Jiang W., Wu Z.M., Yue X.N., Yuan S.J., Lu H.F., Liang B. (2015). Photocatalytic performance of Ag_2_S under irradiation with visible and near-infrared light and its mechanism of degradation. RSC Adv..

[15] Sadovnikova S.I., Kozlova E.A., Gerasimov E.Y., Rempel A.A. (2017). Photocatalytic hydrogen evolution from aqueous solutions on nanostructured Ag_2_S and Ag_2_S/Ag. Catal. Commun..

[16] Di L.J., Yang H., Xian T., Liu X.Q., Chen X.J. (2019). Photocatalytic and Photo-Fenton Catalytic Degradation Activities of Z-Scheme Ag_2_S/BiFeO_3_ Heterojunction Composites under Visible-Light Irradiation. Nanomaterials.

[17] Zhao W., Dai B.L., Zhu F.X., Tu X.Y., Xu J.M., Zhang L.L., Li S.Y., Leung D.Y.C., Cheng S. (2018). A novel 3D plasmonic p-n heterojunction photocatalyst: Ag nanoparticles on flower-like p-Ag_2_S/n-BiVO_4_ and its excellent photocatalytic reduction and oxidation activities. Appl. Catal. B Environ..

[18] Khanchandani S., Srivastava P.K., Kumar S., Ghosh S. (2014). Band Gap Engineering of ZnO using Core/Shell Morphology with Environmentally Benign Ag_2_S Sensitizer for Efficient Light Harvesting and Enhanced Visible-Light Photocatalysis. Inorg. Chem..

[19] Zhao X.X., Yang H., Li R.S., Cui Z.M., Liu X.Q. (2019). Synthesis of heterojunction photocatalysts composed of Ag_2_S quantum dots combined with Bi_4_Ti_3_O_12_ nanosheets for the degradation of dyes. Environ. Sci. Pollut. Res..

[20] Liu Y.P., Geng P., Wang J.X., Yang Z.S., Lu H.D., Hai J.F., Lu Z.H., Fan D.Y., Li M. (2018). In-situ ion-exchange synthesis Ag_2_S modified SnS_2_ nanosheets toward highly photocurrent response and photocatalytic activity. J. Colloid Interface Sci..

[21] Qiu X.P., Yu J.S., Xu H.M., Chen W.X., Hu W., Bai H.Y., Chen G.L. (2016). Interfacial effect of the nanostructured Ag_2_S/Co_3_O_4_ and its catalytic mechanism for the dye photodegradation under visible light. Appl. Surf. Sci..

[22] Xue B., Jiang H.Y., Sun T., Mao F., Ma C.C., Wu J.K. (2018). Microwave-assisted one-step rapid synthesis of ternary Ag/Ag_2_S/g-C_3_N_4_ heterojunction photocatalysts for improved visible-light induced photodegradation of organic pollutant. J. Photochem. Photobiol. A.

[23] Zhang H.L., Wei B., Zhu L., Yu J.H., Sun W.J., Xu L.L. (2013). Cation exchange synthesis of ZnS-Ag_2_S microspheric composites with enhanced photocatalytic activity. Appl. Surf. Sci..

[24] Gao H.J., Wang F., Wang S.F., Wang X.X., Yi Z., Yang H. (2019). Photocatalytic activity tuning in a novel Ag_2_S/CQDs/CuBi_2_O_4_ composite: Synthesis and photocatalytic mechanism. Mater. Res. Bull..

[25] Tian J., Yan T.J., Qiao Z., Wang L.L., Li W.J., You J.M., Huang B.B. (2017). Anion-exchange synthesis of Ag_2_S/Ag_3_PO_4_ core/shell composites with enhanced visible and NIR light photocatalytic performance and the photocatalytic mechanisms. Appl. Catal. B Environ..

[26] Yu C.L., Wei L.F., Zhou W.Q., Dionysiou D.D., Zhu L.H., Shu Q., Liu H. (2016). A visible-light-driven core-shell like Ag_2_S@Ag_2_CO_3_ composite photocatalyst with high performance in pollutants degradation. Chemosphere.

[27] Hu X.L., Li Y.Y., Tian J., Yang H.R., Cui H.Z. (2017). Highly efficient full solar spectrum (UV-vis-NIR) photocatalytic performance of Ag_2_S quantum dot/TiO_2_ nanobelt heterostructures. J. Ind. Eng. Chem..

[28] Aazam E.S. (2014). Photocatalytic oxidation of methylene blue dye under visible light by Ni doped Ag_2_S nanoparticles. J. Ind. Eng. Chem..

[29] Tian Y., Zhou W.W., Tang H.Q., Fu H.B., Wang L.G. (2015). Heterostructure of AuAg nanoparticles tipping on Ag_2_S quantum tubes. Chem. Commun..

[30] Sadovnikov S.I., Kozlova E.A., Gerasimov E.Y., Rempel A.A., Gusev A.I. (2017). Enhanced photocatalytic hydrogen evolution from aqueous solutions on Ag_2_S/Ag heteronanostructure. Int. J. Hydrog. Energy.

[31] Meng Z.D., Ghosh T., Zhu L., Choi J.G., Park C.Y., Oh W.C. (2012). Synthesis of fullerene modified with Ag_2_S with high photocatalytic activity under visible light. J. Mater. Chem..

[32] Hu W.D., Zhao L.H., Zhang Y.T., Zhang X., Dong L.Z., Wang S.S., He Y.M. (2016). Preparation and photocatalytic activity of graphene-modified Ag_2_S composite. J. Exp. Nanosci..

[33] Meng Z.D., Sarkar S., Zhu L., Ullah K., Ye S., Oh W.C. (2014). Facile Preparation of Ag_2_S-CNT Nanocomposites with Enhanced Photo-catalytic Activity. J. Korean Chem. Soc..

[34] Cen C.L., Zhang Y.B., Liang C.P., Chen X.F., Yi Z., Duan T., Tang Y.J., Ye X., Yi Y.G., Xiao S.Y. (2019). Numerical investigation of a tunable dual-band metamaterial perfect absorber consisting of two-intersecting graphene nanorings arrays. Phys. Lett. A.

[35] Zhang Y.B., Cen C.L., Liang C.P., Yi Z., Chen X.F., Li M.W., Zhou Z.G., Tang Y.J., Yi Y.G., Zhang G.F. (2019). Dual-band switchable terahertz metamaterial absorber based on metal nanostructure. Results Phys..

[36] Yi Z., Huang J., Cen C.L., Chen X.F., Zhou Z.G., Tang Y.J., Wang B.Y., Yi Y.G., Wang J., Wu P.H. (2019). Nanoribbon-ring cross perfect metamaterial graphene multi-band absorber in THz range and the sensing application. Results Phys..

[37] De Volder M.F.L., Tawfick S.H., Baughman R.H., Hart A.J. (2013). Carbon Nanotubes: Present and Future Commercial Applications. Science.

[38] Yi Z., Liang C.P., Chen X.F., Zhou Z.G., Tang Y.J., Ye X., Yi Y.G., Wang J.Q., Wu P.H. (2019). Dual-band plasmonic perfect absorber based on graphene metamaterials for refractive index sensing application. Micromachines.

[39] Wang X.X., Zhu J.K., Tong H., Yang X.D., Wu X.X., Pang Z.Y., Yang H., Qi Y.P. (2019). A theoretical study of a plasmonic sensor comprising a gold nano-disk array on gold film with an SiO_2_ spacer. Chin. Phys. B.

[40] Wang X.X., Zhu J.K., Wen X.L., Wu X.X., Wu Y., Su Y.W., Tong H., Qi Y.P., Yang H. (2019). Wide range refractive index sensor based on a coupled structure of Au nanocubes and Au film. Opt. Mater. Express.

[41] Tong H., Xu Y.Q., Su Y.W., Wang X.X. (2019). Theoretical study for fabricating elliptical subwavelength nanohole arrays by higher-order waveguide-mode interference. Results Phys..

[42] Li H.T., Kang Z.H., Liu Y., Lee S.T. (2012). Carbon nanodots: Synthesis, properties and applications. J. Mater. Chem..

[43] Wang X.X., Bai X.L., Pang Z.Y., Zhu J.K., Wu Y., Yang H., Qi Y.P., Wen X.L. (2019). Surface-enhanced Raman scattering by composite structure of gold nanocube-PMMA-gold film. Opt. Mater. Express.

[44] Czech B., Buda W., Pasieczna-Patkowska S., Oleszczuk P. (2015). MWCNT-TiO_2_-SiO_2_ nanocomposites possessing the photocatalytic activity in UVA and UVC. Appl. Catal. B: Environ..

[45] Tahir M.B., Nabi G., Iqbal T., Sagir M., Rafique M. (2018). Role of MoSe_2_ on nanostructures WO_3_-CNT performance for photocatalytic hydrogen evolution. Ceram. Int..

[46] Mahdiani M., Soofivand F., Ansari F., Salavati-Niasari M. (2018). Grafting of CuFe_12_O_19_ nanoparticles on CNT and graphene: Eco-friendly synthesis, characterization and photocatalytic activity. J. Clean. Prod..

[47] Zhao X.X., Yang H., Cui Z.M., Yi Z., Yu H. (2019). Synergistically enhanced photocatalytic performance of Bi_4_Ti_3_O_12_ nanosheets by Au and Ag nanoparticles. J. Mater. Sci. Mater. Electron..

[48] Jiang D.L., Ma W.X., Xiao P., Shao L.Q., Li D., Chen M. (2018). Enhanced photocatalytic activity of graphitic carbon nitride/carbon nanotube/Bi_2_WO_6_ ternary Z-scheme heterojunction with carbon nanotube as efficient electron mediator. J. Colloid Interface Sci..

[49] Shaban M., Ashraf A.M., Abukhadra M.R. (2018). TiO_2_ Nanoribbons/Carbon Nanotubes Composite with Enhanced Photocatalytic Activity; Fabrication, Characterization, and Application. Sci. Rep..

[50] Di L.J., Yang H., Xian T., Chen X.J. (2018). Construction of Z-scheme g-C_3_N_4_/CNT/Bi_2_Fe_4_O_9_ composites with improved simulated-sunlight photocatalytic activity for the dye degradation. Micromachines.

[51] Xia Y., Li Q., Wu X.F., Lv K.L., Tang D.G., Li M. (2017). Facile synthesis of CNTs/CaIn_2_S_4_ composites with enhanced visible-light photocatalytic performance. Appl. Surf. Sci..

[52] Zheng C.X., Yang H. (2018). Assembly of Ag_3_PO_4_ nanoparticles on rose flower-like Bi_2_WO_6_ hierarchical architectures for achieving high photocatalytic performance. J. Mater. Sci. Mater. Electron..

[53] Wang X., Pang Z.Y., Yang H., Qi Y.P. (2019). Theoretical study of subwavelength circular grating fabrication based on continuously exposed surface plasmon interference lithography. Results Phys..

[54] Cen C.L., Yi Z., Zhang G.F., Zhang Y.B., Liang C.P., Chen X.F., Tang Y.J., Ye X., Yi Y.G., Wang J.Q. (2019). Theoretical design of a triple-band perfect metamaterial absorber in the THz frequency range. Results Phys..

[55] Pooladi M., Shokrollahi H., Lavasani S.A.N.H., Yang H. (2019). Investigation of the structural, magnetic and dielectric properties of Mn-doped Bi_2_Fe_4_O_9_ produced by reverse chemical co-precipitation. Mater. Chem. Phys..

[56] Zheng C.X., Yang H., Cui Z.M., Zhang H.M., Wang X.X. (2017). A novel Bi_4_Ti_3_O_12_/Ag_3_PO_4_ heterojunction photocatalyst with enhanced photocatalytic performance. Nanoscale Res. Lett..

[57] Abdullah H., Kuo D.H. (2015). Facile Synthesis of n-type (AgIn)_x_Zn_2(1−x)_S_2_/p-type Ag_2_S Nanocomposite for Visible Light Photocatalytic Reduction To Detoxify Hexavalent Chromium. ACS Appl. Mater. Interfaces.

[58] Xiao S.N., Zhu W., Liu P.J., Liu F.F., Dai W.R., Zhang D.Q., Chen W., Li H.X. (2016). CNTs Threaded (001) Exposed TiO_2_ with High Activity in Photocatalytic NO Oxidation. Nanoscale.

[59] Zhao X.X., Yang H., Zhang H.M., Cui Z.M., Feng W.J. (2019). Surface-disorder-engineering-induced enhancement in the photocatalytic activity of Bi_4_Ti_3_O_12_ nanosheets. Desalin. Water Treat..

[60] Meng X.C., Zhang Z.S. (2017). Plasmonic Z-scheme Ag_2_O-Bi_2_MoO_6_ p-n heterojunction photocatalysts with greatly enhanced visible-light responsive activities. Mater. Lett..

[61] Yan Q.S., Xie X., Liu Y.G., Wang S.B., Zhang M.H., Chen Y.Y., Si Y.S. (2019). Constructing a new Z-scheme multi-heterojunction photocataslyts Ag-AgI/BiOI-Bi_2_O_3_ with enhanced photocatalytic activity. J. Hazard. Mater..

[62] Wang S.Y., Yang H., Wang X.X., Feng W.J. (2019). Surface disorder engineering of flake-like Bi_2_WO_6_ crystals for enhanced photocatalytic activity. J. Electron. Mater..

[63] Meng X.C., Zhang Z.S. (2015). Facile synthesis of BiOBr/Bi_2_WO_6_ heterojunction semiconductors with High visible-light-driven photocatalytic activity. J. Photochem. Photobiol. A Chem..

[64] Ma D., Wu J., Gao M.C., Xin Y.J., Chai C. (2017). Enhanced debromination and degradation of 2,4-dibromophenol by an Z-scheme Bi_2_MoO_6_/CNTs/g-C_3_N_4_ visible light photocatalyst. Chem. Eng. J..

[65] Ning X.B., Ge S.S., Wang X.T., Li H., Li X.R., Liu X.Q., Huang Y.L. (2018). Preparation and photocathodic protection property of Ag_2_S-TiO_2_ composites. J. Environ. Chem. Eng..

[66] Yan Y.X., Yang H., Yi Z., Li R.S., Wang X.X. (2019). Enhanced photocatalytic performance and mechanism of Au@CaTiO_3_ composites with Au nanoparticles assembled on CaTiO_3_ nanocuboids. Micromachines.

[67] Teoh W.Y., Scott J.A., Amal R. (2012). Progress in Heterogeneous Photocatalysis: From Classical Radical Chemistry to Engineering Nanomaterials and Solar Reactors. J. Phys. Chem. Lett..

[68] Tachikawa T., Fujitsuka M., Majima T. (2007). Mechanistic Insight into the TiO_2_ Photocatalytic Reactions: Design of New Photocatalysts. J. Phys. Chem. C.

